# Effect of oral administration of gabapentin on the minimum alveolar concentration of isoflurane in cats

**DOI:** 10.3389/fvets.2023.1117313

**Published:** 2023-02-14

**Authors:** Hangbin Chen, Huan Yang, Mengqing Li, Haojie Peng, Weibin Guo, Meng Li

**Affiliations:** ^1^Department of Veterinary Clinical Sciences, College of Veterinary Medicine, Nanjing Agricultural University, Nanjing, Jiangsu, China; ^2^Ainuo Blessing Veterinary Hospital, Guangzhou, Guangdong, China

**Keywords:** anesthesia, gabapentin, minimum alveolar concentration (MAC), isoflurane, cat

## Abstract

**Objective:**

To determine if oral gabapentin decreases the minimum alveolar concentration (MAC) of isoflurane in cats.

**Study design:**

Prospective, randomized, blinded, crossover, and experimental study.

**Animals:**

A total of six healthy adult cats (three male, three female) aged 18–42 months, weighing 3.31 ± 0.26 kg.

**Methods:**

Cats were randomly given oral gabapentin (100 mg cat^−1^) or placebo 2 h before starting MAC determination, with the crossover treatment given at least 7 days apart. Anesthesia was induced and maintained with isoflurane in oxygen. Isoflurane MAC was determined in duplicate using an iterative bracketing technique and tail clamp method. Hemodynamic and other vital variables were recorded at each stable isoflurane concentration and were compared between gabapentin and placebo treatments at lowest end-tidal isoflurane concentration when cats did not respond to tail clamping. A paired *t*-test was used to compare normally distributed data, and a Wilcoxon signed-rank test was applied for non-normally distributed data. Significance was set at *p* < 0.05. Data are mean ± standard deviation.

**Results:**

Isoflurane MAC in the gabapentin treatment was 1.02 ± 0.11%, which was significantly lower than that in the placebo treatment (1.49 ± 0.12%; *p* < 0.001), decreasing by 31.58 ± 6.94%. No significant differences were found in cardiovascular and other vital variables between treatments.

**Conclusion and clinical relevance:**

Oral administration of gabapentin 2 h before starting MAC determination had a significant isoflurane MAC-sparing effect in cats with no observed hemodynamic benefit.

## Introduction

Inhalation anesthesia is widely used in veterinary practice and is characterized by quick onset, rapid recovery, and adjustability of anesthetic depth. However, using high concentrations of inhaled anesthetics, such as isoflurane, can cause adverse cardiovascular and pulmonary effects in cats (1). Minimum alveolar concentration (MAC) is the most commonly used measure of potency for inhaled anesthetics (2, 3). A decrease in MAC may decrease the concentration of inhaled anesthetics required to maintain adequate depth of anesthesia, which may reduce adverse cardiovascular and pulmonary effects (4, 5).

Gabapentin, 1-(aminomethyl) cyclohexane acetic acid, is a structural analog of gamma-aminobutyric acid (GABA). Although GABA is one of the inhibitory neurotransmitters of the mammalian central nervous system (6), gabapentin does not act on GABA receptors but selectively inhibits voltage-gated calcium channels containing the α2δ-1 subunit (7). Originally, gabapentin was used as antiepileptic therapy to reduce partial seizures (8). In later studies, the effect of gabapentin in treating chronic pain in cats has been revealed (9). More recently, due to its ease of administration and availability, gabapentin is widely used as an anxiolytic in the clinical setting for cats. In addition, oral gabapentin has been described to attenuate fear responses in community cats (10), stimulate appetite for post-ovariectomy (11), contribute to reducing stress during transportation as well as improve compliance in veterinary examination (12). Commonly used oral single doses of gabapentin are 50, 100, and 150 mg cat^−1^, or ~10–30 mg kg^−1^, which caused sedative effects for most cats (10, 12, 13). Pharmacokinetic studies of oral gabapentin in cats have shown that the time to reach maximum plasma concentration was ~1–2 h after a single oral dose of gabapentin (10 mg kg^−1^) (14, 15); the time from oral administration to achieve mild sedative effect was also within this time frame (12, 13).

Perorally administered gabapentin (20 mg kg^−1^) 2 h before general anesthesia maintained with isoflurane was shown to have a MAC-sparing effect in dogs (16). However, intravenous gabapentin administration was reported to have no detectable effects on MAC_ISO_ in cats (17). To the authors' knowledge, no clinical studies have been published assessing the effect of perorally administered gabapentin on the MAC_ISO_ in cats.

The objective of this study was to determine the effect of prior perorally administered 100 mg gabapentin on MAC_ISO_ in cats. We hypothesized that prior perorally administered gabapentin would decrease MAC_ISO_ in cats.

## Materials and methods

### Animals

A group of six purpose-bred cats (three neutered males, one spayed female, and two sexually intact females) aged 18–42 months old, weighing 3.31 ± 0.26 kg [mean ± standard deviation (SD)] were included in this study. The sample size was determined based on previous MAC_ISO_ studies in cats (17–20). Six cats could provide a statistical power of 0.9 to detect a 20% difference in MAC with a 95% confidence interval (17). The cats were determined to be healthy based on physical examination, complete blood count, serum biochemical analysis, and echocardiographic examination. The study was approved by the Institutional Animal Care and Use Committee of Nanjing Agricultural University (20220510100). Food but not water was withheld from cats for 12 h before the experiments.

### Study design and treatments

**Figure 1:**
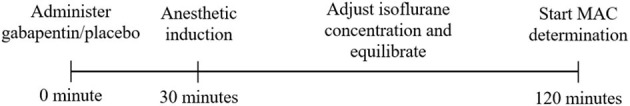
Study timeline. After 30 min of oral administration of the gabapentin capsule or empty capsule, anesthesia was induced in all cats. Isoflurane concentration was adjusted to maintain a light surgical depth of anesthesia and cats were allowed to equilibrate at that concentration at least 120 min after gabapentin/placebo administration. Then, MAC determination was started.

The study was conducted as a prospective, randomized, blinded, crossover trial. Each cat was randomized to receive a 100 mg gabapentin capsule or empty capsule orally 30 min prior to anesthesia induction. The dose of gabapentin was chosen based on prior studies and clinical use of 100 mg gabapentin as sedative dose in cats (10, 12, 13). MAC determination was started 2 h after administration of gabapentin capsule or empty capsule (Figure 1). Then, the crossover trial was implemented after a washout period of at least 7 days. The randomization protocol was obtained using Research randomizer (https://www.randomizer.org/) to generate 6 sets of numbers, and each set had two numbers (0 or 1) to indicate the treatment (placebo or gabapentin). Every cat in the gabapentin treatment was orally administered 100 mg gabapentin (Jiangsu Nhwa Pharmaceutical Co., Jiangsu, China), and cats in the placebo treatment were orally administered empty capsules. After gabapentin or empty capsules administration, 2 mL of water was given with a syringe.

### Anesthesia and instrumentation

A 24-gauge catheter was aseptically placed in the cephalic vein. An integrated anesthesia machine with ventilator (Dräger Vapor^®^ 2000; Drägerwerk AG & Co., Germany) was used for the experiment. Anesthesia was induced with 5% isoflurane (Jiangsu H.F.Q Bio-technology Co., Jiangsu, China) delivered *via* a face mask with oxygen at a flow rate of 5 L min^−1^. Following loss of jaw tone, 0.1 mg kg^−1^ lidocaine (Shandong Hualu Pharmaceutical Co., Shandong, China) was topically applied to the arytenoid cartilages. Then the trachea was intubated with an appropriately sized and cuffed endotracheal tube. A gas sampling connector was placed between the endotracheal tube and the Y-piece of a circle breathing system. Then a catheter was positioned within the lumen of the gas sampling tube and the endotracheal tube and its tip was in proximity to the distal (animal) end of the endotracheal tube. All experiments were conducted at sea level. End-tidal isoflurane concentration (FE′Iso) and end-tidal partial pressure of carbon dioxide (PE′CO_2_) were monitored with a calibrated side-stream (gas sampling rate: 50 mL min^−1^) infrared gas analyzer (AG module; Mindray, Shenzhen, China).

Cats were positioned in right lateral recumbency, and anesthesia was maintained with isoflurane in oxygen with a flow rate of 1 L min^−1^ delivered *via* a circle breathing system. Light surgical depth of anesthesia was maintained by adjusting isoflurane concentration (21). Cats were mechanically ventilated using volume-controlled ventilation with a respiratory rate of 15 breaths min^−1^ and tidal volume of 15 mL kg^−1^ min^−1^, adjusting the respiratory rate and tidal volume to maintain PE′CO_2_ between 30 and 45 mmHg. Lactated Ringer's solution was intravenously administered at 3 mL kg^−1^ h^−1^ throughout anesthesia. A forced warm-air blanket was applied to maintain rectal temperature (T) within 38.5–39.5°C, measured continuously using a calibrated thermometer. The non-invasive systolic blood pressure (SBP) was obtained by a Doppler ultrasonic device (Model 811B; Parks medical electronics, Inc., OR, USA) with a Doppler crystal placed over the median artery of the left forelimb and an occluding cuff placed proximally. The cuff size was selected to be ~40% of the circumference of the mid left forelimb or hindlimb. A pulse oximeter probe was placed on the tongue to measure arterial hemoglobin oxygen saturation percentage (SpO_2_). Heart rate (HR), simultaneous electrocardiogram (ECG), FE′Iso, PE′CO_2_, and SpO_2_ were continuously monitored with a multi-parameter monitor (iPM12 Vet; Mindray, Shenzhen, China). All variables were monitored throughout and were recorded at the end of every equilibration period.

### MAC_ISO_ determination

The MAC was determined in duplicate using the bracketing technique and tail clamping method as previously described (5, 21, 22). A noxious stimulus was applied using a 20-cm Martin forceps positioned on the base of the tail and closed to the first ratchet. Stimulation ceased if cats showed movements or no movements for a minute of tail clamping. Movements were defined as twisting, jerking of the head, running, or clawing movements of the limbs; while coughing, swallowing, or chewing were not considered positive responses (2, 23–27). The determination of whether there was a positive reaction or not was always made by an investigator who was blinded to treatment.

FE′Iso was kept constant for at least 15 min prior to each MAC determination. The starting isoflurane concentration was set between 1 and 2% depending on the treatment, a noxious stimulus was applied and the cat was observed for a positive or negative response (21). Isoflurane concentration was decreased or increased by 10% each time after a negative or positive response to tail clamping, respectively. This procedure was repeated until two successive FE′Iso were detected, one allowing a positive response and one preventing a positive response. The first MAC_ISO_ was the mean value of these two successive FE′Iso. Next, the MAC was determined a second time. Here, the starting point was set at 0.2% higher than the previous highest FE′Iso at which a positive response was observed. The MAC_ISO_ was reported as the mean value of the two MAC values.

When the MAC_ISO_ determination was achieved, the isoflurane administration was discontinued and the cats were maintained on ventilator with oxygen delivered at 1 L min^−1^ until extubation. The FE′Iso at which the cat could not tolerate tracheal intubation (exhibiting signs of coughing or swallowing) was recorded and the endotracheal tube was removed. The time from discontinuing isoflurane administration to tracheal extubation was recorded at the same time.

### Statistical analysis

Data were analyzed using SPSS Statistics 26.0 (IBM Corp., CA, USA) and visualized using GraphPad Prism 9.2.0 (GraphPad Software, CA, USA). Normality was assessed with a Shapiro-Wilk test and normally distributed data are presented as mean ± SD. Non-normally distributed data are reported as median [range]. A two-tailed paired *t*-test was used to determine the difference between the gabapentin treatment and the placebo treatment for normally distributed data, and a Wilcoxon signed-rank test was used for non-normally distributed data or unequal amounts of data between treatments. The significance was set at *p* < 0.05.

## Results

**Figure 2:**
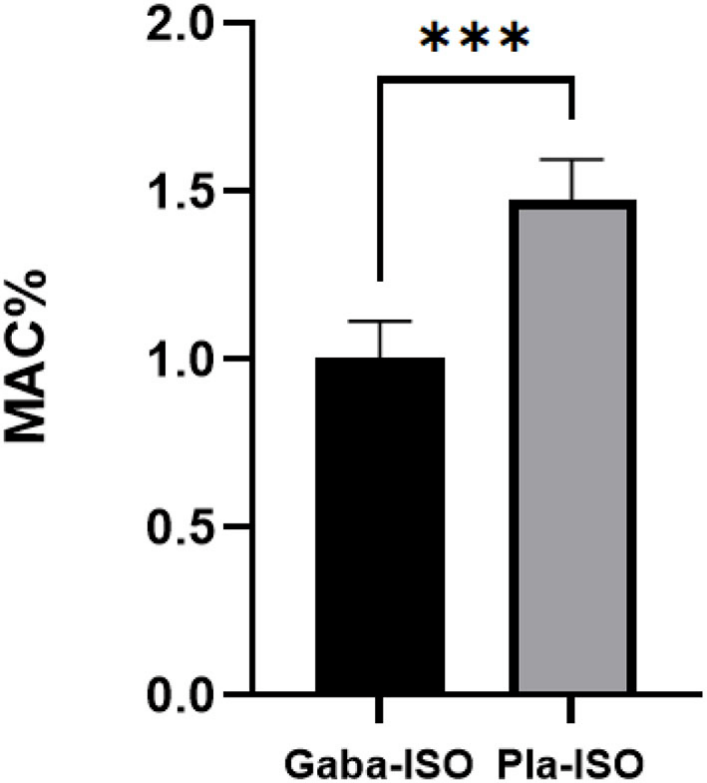
Minimum alveolar concentration of isoflurane in the gabapentin and placebo treatments. Gaba-ISO, six cats were orally administered with 100 mg gabapentin 2 h before starting MAC determination and then maintained with isoflurane; Pla-ISO, six cats were orally administered with empty capsules 2 h before starting MAC determination and then maintained with isoflurane. ***Significant difference between two treatments (*p* < 0.001).

Six cats weighing 3.31 ± 0.26 kg were perorally administered 100 mg gabapentin, resulting in a dosage of 30.36 ± 2.46 mg kg^−1^ in the gabapentin treatment. The MAC_ISO_ of the gabapentin treatment was 1.02 ± 0.11%, which was significantly (*p* < 0.001) lower than in the placebo treatment (1.49 ± 0.12%; Figure 2). The MAC_ISO_ decreased by 31.58 ± 6.94% with prior administration of gabapentin.

When equilibration of FE′Iso was reached throughout anesthesia regardless of responses to tail clamping, SBP was 118 ± 24 mmHg for all measurements. T (all measurements pooled) was 38.8 ± 0.2°C. PE′CO_2_ (all measurements pooled) was 34 ± 4 mmHg. After equilibration at the lowest FE′Iso, when cats did not respond to tail clamping, no significant differences were found between the gabapentin treatment and the placebo treatment in cardiovascular and other vital variables (Table 1). The FE′Iso at extubation in gabapentin treatment was 0.25 ± 0.05%, which was significantly (*p* = 0.005) lower than that in the placebo treatment (0.48 ± 0.10%). No significant differences in the time from discontinuation of isoflurane to extubation were found between gabapentin and placebo treatments.

**Table 1:** Values for cardiovascular and other vital variables for six cats anesthetized with isoflurane after oral administration of gabapentin (100 mg cat^−1^) or empty capsules 2 h before starting MAC determination; cardiovascular and other vital variables were obtained after equilibration at the lowest FE′Iso when cats did not respond to tail clamping.

**Variables**	**Gabapentin treatment**	**Placebo treatment**
PE′CO_2_ (mmHg)	33 (30–39)	34 (30–39)
SpO_2_ (%)	100 (98–100)	100 (100–100)
HR (beats minute^−1^)	189 (119–217)	187 (109–225)
SBP (mmHg)	115 ± 23	120 ± 27
T (°C)	38.8 (38.7–39.2)	38.9 (38.6–39.1)
FE′Iso (%) at extubation	0.25 ± 0.05	0.59 ± 0.10^*^
TE (minutes)	7 ± 4	6 ± 4

PE′CO_2_, end-tidal partial pressure of carbon dioxide; SpO_2_, arterial hemoglobin oxygen saturation percentage; HR, heart rate; SBP, systolic blood pressure by Doppler ultrasonic device; T, rectal temperature; FE′Iso, end-tidal isoflurane concentration; TE, time from discontinuation of isoflurane to extubation.

^*^Significant difference between two treatments (p < 0.05).

The time from gabapentin/placebo administration to starting MAC determination was 122 ± 2 min in gabapentin and placebo treatments, with no significant differences found between treatments. The duration of anesthesia and the time from gabapentin/placebo administration to the first and second MAC determination were significantly less in the placebo treatment than those in the gabapentin treatment (*p* = 0.012, *p* < 0.001, *p* = 0.018, respectively; Table 2).

**Table 2:** Time from gabapentin/placebo administration to starting MAC determination, the first and second MAC determination.

**Variables**	**Gabapentin treatment**	**Placebo treatment**
TS (minutes)	122 ± 2	122 ± 2
T_I_ (minutes)	196 ± 20	166 ± 22^*^
T_II_ (minutes)	262 ± 19	215 ± 24^*^
DA (minutes)	219 ± 20	173 ± 24^*^

TS, time from gabapentin/placebo administration to starting MAC determination; T_I_, time from gabapentin/placebo administration to the first MAC determination; T_II_, time from gabapentin/placebo administration to the second MAC determination; DA, duration of anesthesia.

^*^Significant difference between two treatments (p < 0.05).

## Discussion

This study supports our hypothesis that prior perorally administered gabapentin would decrease MAC_ISO_ in cats. Gabapentin (100 mg cat^−1^) perorally administered 2 h before starting MAC determination maintained with isoflurane significantly reduced MAC_ISO_ by 31.58 ± 6.94% compared with the placebo treatment. This finding is in agreement with a previous study in dogs that oral gabapentin (20 mg kg^−1^) had a 20 ± 14% MAC-sparing effect (16). The MAC_ISO_ range for cats was reported to be between 1.20 ± 0.13% and 2.22 ± 0.35%, while the mean MAC_ISO_ in cats was 1.71 ± 0.07% (28). In this study, the MAC_ISO_ for the placebo treatment (1.49 ± 0.12%) is within the reported range, but slightly lower than the reported mean MAC_ISO_. The FE′Iso at extubation in gabapentin treatment was significantly lower than that in the placebo treatment. Possibly due to the MAC-sparing effect of gabapentin, cats were allowed to tolerate the endotracheal tube at a lower FE′Iso in the gabapentin treatment. But the significance of FE′Iso at extubation is difficult to interpret because it was obtained without equilibration, therefore not representative of the partial pressure in the central nervous system. PE′CO_2_ and body temperature were both kept within the target range. Although there was no significant difference in cardiovascular variables between gabapentin and placebo treatments, implying that gabapentin may not bring significant hemodynamic benefits in anesthetized cats, the results should be interpreted with caution due to the small sample size. A recent study examined the effects of oral gabapentin (27.9 ± 2.6 mg kg^−1^) within 2 h of administration on sedation, hemodynamic and echocardiographic variables in awake cats. Heart rate and blood pressure did not differ significantly between the baseline, placebo, and gabapentin treatments, while most cats exhibited signs of sedation with mild ataxia and had a reduction in echocardiographic systolic function albeit they remained within the reference range (13). Gabapentin may have limited effects on hemodynamic variables in either anesthetized or awake cats, but further investigations are warranted for verification.

The exact mechanism by which gabapentin reduces MAC remains to be determined. Studies have shown that gabapentin provides an antinociceptive effect by binding to the alpha2-delta subunit of voltage-gated calcium channels, which reduces the release of glutamate, norepinephrine (noradrenaline), and substance P (29). However, it remains unknown whether the MAC-sparing effect of gabapentin is due to its antinociceptive effect (30).

The effect of gabapentin on MAC differs based on animal species and routes of administration. In a study evaluating the effects of intraperitoneal gabapentin treatment on MAC_ISO_ in adult male rats, only high dosages of intraperitoneal gabapentin were shown to significantly reduce MAC (31). When gabapentin was administered intraperitoneally at concentrations of 300 and 1,000 mg kg^−1^, MAC_ISO_ was significantly reduced by 19 and 18%, respectively (31). The effects of intravenous gabapentin administration on the MAC_ISO_ in cats have been investigated, but no significant differences were identified at any of the target plasma concentrations (17). This finding contradicts our findings and previous studies in dogs and rats (16, 31). The cause for the difference in MAC-sparing effect between oral and intravenous administration of gabapentin is unclear, and additional study is warranted to investigate the effect and mechanism of different gabapentin administration routes on MAC in cats.

There are certain limitations in the current study. Lidocaine was used prior to tracheal intubation and may have been systemically absorbed to reduce MAC (32). However, due to the low dose as well as the long duration between lidocaine administration and MAC determination, the effect of lidocaine on MAC may be marginal. The statistical power for detecting effects on cardiovascular and other vital variables is unknown, but it is probably low because the sample size was chosen according to the power analysis to identify changes in MAC_ISO_. Besides, the cardiovascular effects of gabapentin in anesthetized cats were not designed to explore in detail, so the cardiovascular effects of gabapentin warrant additional investigation. Positive pressure ventilation was used during anesthesia which might decrease cardiac output (33), and affect blood pressure. Due to the invasiveness and technical challenges, non-invasive blood pressure (Doppler) rather than arterial blood pressure measurement was employed in this study. Doppler has been shown to underestimate systolic blood pressure in anesthetized cats (34), therefore, SBP reported in this study may not represent its true systolic blood pressure. Based on study timeline, the equilibration time before the first noxious stimulation was longer than in other similar studies (21, 23). Owing to fewer adjustments of isoflurane concentration in the placebo treatment, the duration of anesthesia and the time from gabapentin/placebo administration to the first and second MAC determination were less in the placebo treatment than in the gabapentin treatment. However, the duration of anesthesia is not known to affect MAC_ISO_ (35). In this study, a single-point calibration was used which might affect the accuracy and reliability of the MAC determinations (36). Gabapentin plasma concentrations were not measured concurrently, making it difficult to evaluate the relationship between plasma concentrations and MAC determinations. Two pharmacokinetic studies of single oral administration of gabapentin in cats agreed that a 1-compartment model with lag time fitted best for the decrease in the plasma concentration of gabapentin after oral administration, and peak plasma concentrations of single oral doses of gabapentin (10 mg kg^−1^) were 7.982 ± 1.053 and 12.42 (8.31–18.35) μg mL^−1^ after 100 ± 22 and 63 (44.4–126.6) min of administration, respectively (14, 15). However, the mean time from drug administration to MAC determination was about 240 min, at which time gabapentin plasma concentrations had declined from peak concentrations. In future studies, simultaneous analysis of gabapentin plasma concentration is still needed, although plasma concentrations may or may not represent target tissue concentration (central nervous system in this case).

## Conclusion

In conclusion, this study found that oral administration of 100 mg gabapentin 2 h before starting MAC determination reduced MAC_ISO_ with no observed hemodynamic benefit. Further studies of other formulations of gabapentin and variable dosing regimens and their correlation with plasma concentration and MAC_ISO_ in cats are warranted.

## Data availability statement

The original contributions presented in the study are included in the article/supplementary material, further inquiries can be directed to the corresponding author.

## Ethics statement

The animal study was reviewed and approved by Institutional Animal Care and Use Committee of Nanjing Agricultural University.

## Author contributions

HC: study design, statistical analysis, and preparation of manuscript. HY: study design, data acquisition, and preparation of manuscript. MengqL: study design and statistical analysis. HP: study design and data acquisition. WG: study design and preparation of manuscript. MengL: study design, data management, and preparation of manuscript. All authors approved the final version of the manuscript.
